# Integrating Interactive Clothing and Cyber-Physical Systems: A Humanistic Design Perspective

**DOI:** 10.3390/s20010127

**Published:** 2019-12-24

**Authors:** Weizhen Wang, Yuan Fang, Yukari Nagai, Dong Xu, Tsutomu Fujinami

**Affiliations:** 1 National Demonstration Center for Experimental Fashion Design and Engineering Education, Dalian Polytechnic University, Dalian 116034, China; 2 School of Information Science and Engineering, Dalian Polytechnic University, Dalian 116034, China; 3 Japan Advanced Institute of Science and Technology, Nomi 923-1292, Ishikawa, Japan; 4 School of Arts, Tianjin Polytechnic University, Tianjin 300387, China

**Keywords:** interactive clothing, cyber-physical systems, cyber-physical-clothing systems, humanistic evaluation, clothing design, autism

## Abstract

This study is aimed at bridging the gap from a transdisciplinary perspective between cyber-physical systems (CPS) architecture in the field of information science and emotional design in the field of humanistic science for interactive fashion innovation. Information related to a familiar feeling in the process of interactive clothing design is used to explain how the transformation could be realized from data. By creating the cyber-physical-clothing systems (CPCS), the architecture model in the hyper world and takes the development process of an interactive parent-child clothing as a case study for analyzing the transformation from the physical signal input to the social symbol recognition output. The experimental results, which from the perspective of clothing art design rather than information discipline, show that interactive parent-child clothing is not only suitable for the rehabilitation of autistic children but also recognized by most parents. The reasonable embedding of sensing technology can greatly enhance the added value of clothing products. This study provides a fruitful practical application reference for designers who are engaged in the field of art and design but not familiar with the relevant information technology. Furthermore, the application principle and the technical process of CPCS for further interactive clothing design is explained.

## 1. Introduction

As a multi-dimensional smart mechanism that tightly integrates computing, communication, and control [1,2] with a deep interaction between the physical and cyber world, cyber-physical systems (CPSs) provide a human computer interface (HCI) with the foundation of emerging and future smart services and improve our quality of life in many areas [3,4]. The smart clothing for physiotherapy rehabilitation and sports training monitoring is one of the important applications [5,6,7]. Following the ascending application of CPS to the fashion industry, the academic and industry research concerns in this area across various disciplines stems have mainly been devoted to optimizing the interaction of smart clothing through the integration of information technology. Therefore, as a development branch of smart clothing and also one symbolic medium in the field of sociology [8], interactive clothing, a new concept was first proposed in our previous research, emphasizes the interactive characteristics of communication and expression between human and objects [9], and it is adopted in this study as a target vector for autism rehabilitation.

In recent years, CPS has been adopted by an increasing number of product development brands after their effectiveness was shown in the 2017 and 2018 Milan, Paris, and New York Fashion Week shows. Undeniably, technological innovation can enhance a fashion brand’s marketing campaign and catch the attention of consumers. CPS in the future will be integrated into our clothing for daily life [10,11,12].

However, several challenges still exist from the perspectives of correlative information technology and humanities design. The purpose of this case study is to rise to the following two challenges by bridging the gap between CPS in the field of information science with art and design or the emotional evaluation in the field of humanistic science.

Firstly, the existing research on smart clothing is limited to the physiological characteristics of perception and feedback but neglects the social interaction function of clothing. It is a significant challenge to apply CPS in the development of interactive clothing when the functional definition of interactive clothing is extended to provide the presentation of interactions between different wearers, especially autism groups, rather than just personal interaction with his body data [13,14]. That is, how to meet the differentiated needs of different wearers and the differences in various attributes among each wear group.

Secondly, the development of interactive clothing lacks the guidance of a theoretical system model, and clothing designers engaged in art design do not know how to use sensor technology to achieve the automatic interaction effect of clothing to trigger the communication desire of autistic patients. Current researchers seldom dabble in the systematic research of smart clothing from information modeling to humanistic evaluation, and most of them analyze the information technology application or humanistic design respectively from a single academic background [15,16,17,18,19], which may lead their inability to systematically explain the principle and process of how to extract data as well as transform and form a humanistic feeling of the interactive clothing.

Two questions are answered to attain the above-stated objectives. Q1: How to dismantle the CPS architecture so that it can be effectively applied to the design and development process of interactive clothing? Q2: How can CPS technology be adopted to achieve the multiple interactions between the dresser especially those with communication disorders and their environment?

This study expanded and subdivided the CPS architecture of interactive clothing and conducts the Kansei Engineering [20] analysis of wearers’ evaluation to understand and expound interactive design from the perspective of humanities, rather than developing or validating it from the perspective of information science. Furthermore, the R&D elements are explored and practical implications for interactive clothing design are suggested.

Our contributions in this paper are

- Proposes and defines the concept of one new branch of smart clothing, which is interactive clothing, and take it as the carrier to study the feasibility of rehabilitation treatment for autistic children.
- Create an innovative cyber-physical-clothing systems (CPCS) model and analyses its feasibility with the interactive parent–child clothing development as a case.
- This study understands and expounds interactive design from the perspective of humanities design rather than developing or validating it from the perspective of information science. It also adopts the method of humanities design to explain how to integrate interactive clothing and CPS, provides an application reference on interactive design for non-information engineers.

## 2. Definition of Interactive Clothing

The research subject of this study refers to the interactive clothing, which is a new category of smart clothing in the field of information science. The concept where an interactive balance was sought between the wearers and their environment based on the integration of information science and traditional clothing, rather than the biological material science category of bio-smart clothing [21,22].

Interactive clothing originated from wearable devices, and it is a development branch of smart clothing. In addition to the special information science function of self-monitoring analysis and reporting technology that are owned by smart clothing and the basic physical functions of traditional clothing, interactive clothing places more emphasizes on clothing that can carry the media function of society and humanities in the process of interactive activities between people and the environment based on information technology. By combining CPS with clothing engineering design to input a certain physical signal into clothing, so the clothing can output a specific social symbol that people or the clothing environment can perceive to generate the corresponding interaction. This kind of social symbol is the expression form of the clothing as the interactive medium [23].

Researchers should take the human body as a critical frontier for creating compelling and deeply engaging ways of interacting with computers [10,24]. As a symbolic concept clothing, the parent–child clothing is a perfect symbolic carrier of the emotional design of clothing, which can create a micro-hyper world to express the integration of family belonging or clothing culture and emphasizes interaction in everyday family life and the family circle’s emotional relations [25,26] that are the elements that are lacking in children with autism spectrum disorders [27]. As Jeon [28] advocated emotions and affect in human computer interaction, interactive parent–child clothing conforms to the CPS’ technical conditions in which the interpersonal signal output interacts with the social symbol feedback of the family atmosphere. Therefore, interactive parent–child clothing can integrate behavior therapy, play therapy, and sensory integration therapy to carry out a natural treatment for children with autism spectrum disorders [29,30].

## 3. Creating the CPCS Model

As there is no relevant theoretical model to provide an appropriate reference for art designers, we intend to build a basic technical framework model to provide product creation ideas.

**Figure 1:**
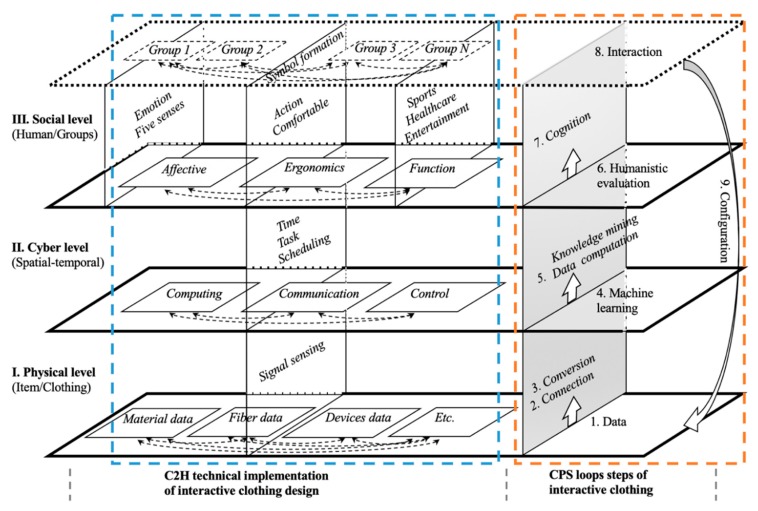
CPCS model.

Bizopoulos and Koutsouris [31] advocated the creation of complex theoretical models not only in view of its structure and function but also in the refinement for application. According to the 5C architectural model [32,33], the CPS could provide conceptual guidance for the development of interactive clothing. The technical process of interactive clothing design and development, that is, the technical relevance of interactive clothing and CPS were revealed in the cyber-physical-clothing systems (CPCS) model (Figure 1). The role of this model is to explain the technical development process of interactive clothing from clothing to data, information, knowledge, wisdom, services, humans, and then back to clothing. This is called the clothing to the human cycle (C2H) from the perspective of CPS architecture.

Interactive clothing has the characteristics of information processing and human feedback, it is controlled by machine-based algorithms, and it integrates the network with its wearers into one unified holistic framework, which determines its interactive architecture. Like other scholars’ research results, can be divided into three levels: physical, cyber, and social [34,35]. The vertical direction in this model represents the functional relationship of progressive interaction from the physical level to the cyber level and social level, and the horizontal direction corresponds to the technical principle relationship in the three levels between C2H technical implementation (inside the blue dotted box), and CPS loops (inside the red dotted box). The dotted double arrows represent interactions and effects, and the solid lined wide arrows represent development directions. This model fully and clearly responds to the Q1 mentioned above.

### 3.1. Technical Processing of Interactive Clothing at the Physical Level

The entity item of the physical level is clothing (Figure 1).

(1) This level is implemented by embedding the sensors and microprocessors in combination with the style design of the clothing to create a network that performs the data connection from the clothing material or devises, forms special signals, and finally, implements the signal conversion to the cyber level employing sensing.
(2) The characteristic of the process from Step 1 to Step 2 and Step 3 is the informatization process from the basic data of the physical level of clothing to the cyber level.
(3) Among the contents of this process, the process from Step 1 to Step 2 belongs to IntelliSense. The core of this process is the creation of a CPS architecture that allows data to be measured and aggregated in various forms. Besides, communication between embedded sensors and other devices is also possible. The process from Step 2 to Step 3 is information mining. The core of this process is the machine-based algorithms on the device side so that some data can be analyzed and utilized locally in the device to achieve local intelligence.
(4) There are also interactions between different categories of data.

### 3.2. Technical Processing of Interactive Clothing at the Cyber Level

The cyber level represents the spatial-temporal concept of clothing in the process of interactive information processing. It is the information technology processing stage in the process of transforming physical signals into humanistic symbols [36].

(1) The cyber level, which covers Step 4 and Step 5 in the CPS process, is the core of data processing, distribution, decision making, and scheduling control of the entire clothing CPS system. Here, a big data environment originates from the clothing or wearer while advanced analysis algorithms are run for large-scale computing and knowledge mining.
(2) The content of this level is the organic integration and deep cooperation of computing-communication-control utilizing time, task, and scheduling. Deep integration of the cyber and physical world is achieved involving the object mechanism, environment, and group.
(3) The characteristic of this level is the process of transforming the signaling of clothing data into the symbolization of knowledge from the humanities.
(4) Roush [37] and Donath [38] proposed that computing means connecting, and the social machine is aimed to design for living online. Further, according to the findings published in Nature [39], the quantifiable data rules of interpersonal relationships can be summed up by machine learning calculations from many human behavior experiments. As a technical means of data intelligent, in Figure 1, machine learning’s role is mainly to link the two stages of Step 3 and Step 5. Around 3C, it paves the way for time, task, and scheduling in the subsequent technical process, and provides data signal output that can reflect the behavior of the wearer.

### 3.3. Technical Processing of Interactive Clothing at the Social Level

The social level represents the humanities activities, interpersonal interaction, and communication between individuals or groups [33] using interactive clothing as the medium.

(1) This level includes Step 6, Step 7, and Step 8. The main role is to evaluate, identify, and provide feedback on decision-making for the interactive activities of clothes. Interactive decision-making of collaborative optimization among multiple pieces of clothing is established by analyzing the task objectives and status exhibited by each piece of clothing in the current system.
(2) The main content is humanistic evaluation and cognitive formation. At present, the design evaluation indicators for smart clothing or interactive clothing are mainly divided into three dimensions, that is, effective indicators (emotion, five senses, etc.) [40], ergonomics indicators (action, posture, comfort, etc.) [41], and functional indicators (sport monitoring, healthcare, entertainment, etc.) [18,42]. The follow-up prototype evaluation in this study adopted the Kansei evaluation method.
(3) The technical difficulties are the formation of cognition after performing group differentiation evaluation and the guidance of symbolic interactive feedback. Due to the interaction among different wearer groups, it is necessary to meet the differentiated needs of different types of dress groups, as well as the differences in various issues and attributes among each wearer group.

### 3.4. Technical Processing of Interactive Clothing on the Cross-Level Structure

The configuration execution (Step 9) runs through the three levels of physical, cyber, and social. After receiving the decision, the configuration execution layer transforms the decision into instructions that are understood by the logic of each subsystem. Finally, the microprocessor and sensor embedded in the clothing perform some external reaction of the clothing.

## 4. Prototype Design Following the CPCS Model

### 4.1. The Prototype Development Approach

The style design process considered the form of a three-piece family clothing combination of parents and children. An interactive re-design can highlight the significance of the integration between humanities and technology because family clothing is a typical representative of humanistic emotion [9].

According to the CPCS (Figure 1), we intend to design the prototype according to the following technical path: from the physical level of device data extraction, signal sensing, to the cyber level of 3C and time, task, scheduling, and then into the social level of entertainment and healthcare function design, and finally to achieve group interaction. Sensing technology was used to create a family LAN system for three-piece clothing.

The visual effect of an LED is utilized for the appearance of the garment. Because of the three major elements of clothing product design, that is, the style, color, and material belonging to the visual category [43], the LED illumination effect was noticeable [44].

### 4.2. Design Methods of Interactivity

The target wearers of the prototype are the family trio, that is, the father, mother, and child’s three pieces of clothing for one parent–child clothing series, with an interactive effect that transcends traditional parent–child clothing.

Regarding the technical design of the interaction between physical signals and social symbols, from a psychological point of view, the physical space distance can represent a social symbol of intimacy or alienation of interpersonal relationships [45]. Therefore, this study used physical space distance as the signal trigger for interpersonal interaction. If the three pieces of clothing will form a variety of interactions with one another, the specific design ideas, that is, the initial arrangement of the time, task, and scheduling was as follows:

- When the distance between the child and father wearing the prototypes is reduced, their clothing appearance simultaneously produces a pattern of change at the same time.
- When the distance between the child and mother is reduced, their clothing appearance simultaneously produces a change.
- When the child and parents were close to the set distance at the same time, the appearance of the three pieces of clothing simultaneously causes a change.
- When the father and mother are close, their clothing also causes a kind of change.
- Besides, every piece of clothing has its self-reaction.

### 4.3. Material Selection

#### 4.3.1. Selection of New Fabrics

Regarding the clothing fabric, the inner fabric was mainly selected by splicing DuPont Tyvek paper fabric and 3M luminous reflective fabric. The luminous reflective fabric could show different colors in the states of light and no light, which changes the traditional visual concept of clothing. The outer fabric of clothing was a TPU Transparent film which is a class of burning furnace air pollution-free fabrics. The smooth and transparent texture could show a fashionable appearance of high technology and a futuristic style.

#### 4.3.2. Selection of the Network Components and LED Strip

**Figure 2:**
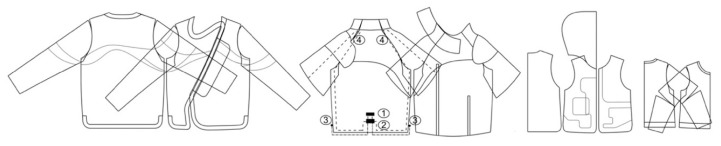
Pattern design and hardware embedding.

Figure 2 shows the pattern design of the three pieces of clothing, from left to right is the father’s clothing, mother’s clothing, and children’s clothing. To illustrate the hardware embedded content, the mother’s clothing can be taken as an example.

**Figure 3:**
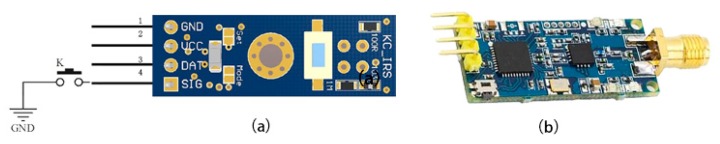
(**a**) KC_IRS, (**b**) DL-22.

To create a network with clothing as an interactive medium, the basic network components embedded in the clothing included power supplies, sensors, chips, and microprocessors. In terms of the electronic components, each piece of clothing independently used a battery (position 1 in Figure 2) and a microcontroller STC8051 (Xinwei Electronic Technology Co., Ltd., Jiaxing, China) (position 2 in Figure 2) as the main control chip to control the sensor response, LED, and communication between the three pieces of clothing. The horizontal KC_IRS (Figure 3a) of Shenzhen Xinyujia Electronic Technology Co., Ltd. (position 3 in Figure 2), was adopted for the sensor selection as an infrared sensor module. We used the crystal oscillator of 11.0592M to provide the clock of the smallest system. The full-duplex UART serial I/O port of the STC8051 microcontroller and the partially parallel I/O port are used as the input and output ports for the digital signal. In terms of communication node design, we used the ARM Cortex-M4 microcontroller (Texas Instruments) and the EK-TM4C123GXL development board (Ji Guan Hui Electronics Co., Ltd., Shenzhen, China) in three UART serial ports as communication nodes to process the signals from three sets of clothing. The DL-22 (Figure 3b, Deep Link Technology Co., Ltd., Shenzhen, China) wireless serial module is used in the three sets of clothing wireless communication scheme.

**Figure 4:**
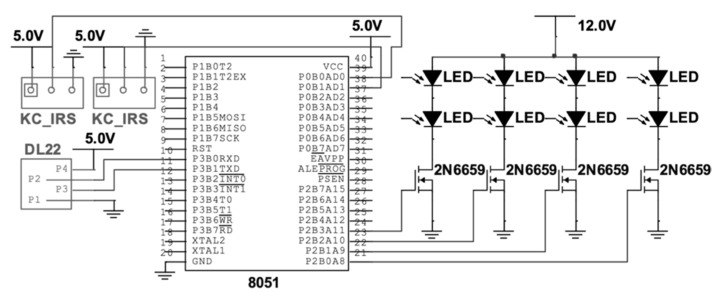
Circuit schematics.

Two types of flexible light strips were selected to be embedded in the clothing for the clothing-mediated manifestation of the social symbol output. The LED using FPC as a base plate could be an arbitrarily curved flexible flat strip with a 5050-60GRB model (Jingzheng Lighting lamp Co., Ltd., Shanghai, China) (position 4 in Figure 2) and can be fixed to a variety of letters or patterns of shape. An EL cold light strip, with the characteristics of repeatedly being folded and curved, low power consumption, and the ability to achieve three kinds of effects (steady-slow, flash-fast, and flash), was suitable for use in clothing design. The circuit schematic is shown in Figure 4.

### 4.4. Prototyping

**Figure 5:**
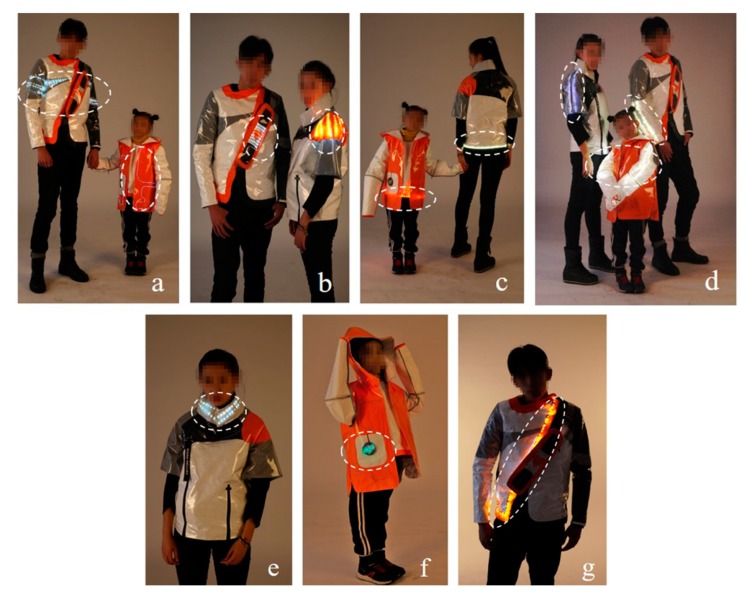
Prototypes. (**a**) The interaction between father’s and child’s clothing; (**b**) Interaction between a husband’s and wife’s clothing; (**c**) Interaction between mother’s and child’s clothing; The interaction between father’s and children’s clothing; (**d**) Interaction between father’s, mother’s, and children’s clothing; (**e**) Self-reaction of mother’s clothing; (**f**) Self-reaction of child ‘s clothing; (**g**) Self-reaction of father’s clothing.

The design and production details of this series of prototypes are shown in Figure 5 [9]. It should be noted that the special effects are indicated by the white dotted circle.

#### 4.4.1. Four Types of Interaction Performances

When the distance between the child and father decreased, the two garments produced a reaction. The performance form is the front of the child’s wearable LED thin-line strips and the father’s clothing horizontal LED strips, which both started shining at the same time (Figure 5a).

When the father and mother stood together, the heart-shaped LEDs pattern on the chest of the father’s clothing and the LEDs pattern of the mother’s shoulder shined simultaneously (Figure 5b).

When the distance between the child and the mother decreased, the two garments produced a reaction. The manifestation is that the LED strips embedded in the hem of their garments started to shine at the same time (Figure 5c).

When the child and parents were close to the set distance at the same time, the sleeves of the three wears shined simultaneously to produce a change (Figure 5d).

#### 4.4.2. Three Types of Self-Interaction Performances

For the mother’s clothing, the embedded LEDs are activated when the collar buckled up, to achieve the expected interactive effect (Figure 5e).

For the child’s clothing, the reaction was through the Kcirs infrared sensor according to the reaction of the hat, which caused causing the front pocket decorative LED to activated and achieved the expected interactive effect (Figure 5f).

For the father’s clothing, the reaction was triggered by the induction of the photosensitive diode module, which caused the front of the zipper part of the LED strips to be activated, which achieved the previous envisaged interactive effect (Figure 5g).

According to the above-stated various demonstration effects of these prototypes, embedding the CPS could greatly enrich the design concept and manifestation of the parent–child clothing. This series of clothing achieves the interaction of individuals and groups as mentioned in the social level of the CPCS model and realizes the representation of the social symbol from the physical distance signal recognition to the parent–child relationship. This simple transformation process from data to information and knowledge illustrates the application forms of CPS in the process of interactive clothing design and development, which response to the aim of this study and Q2 mentioned above.

## 5. Dressing Experiments for Autistic Children

To verify the auxiliary therapeutic effect of the prototype on autistic children, two 15-min comparative experiments were carried out between the wearing status of their own normal daily clothing and the activated interactive prototype we provided. Participants included autistic children and their parents, who were three in two groups. The location is chosen in the public space of the children’s activity centre playroom. The evaluation indicator was language expression, interpersonal communication, behavior, interest expression, and perception communication (Table 1). The scores were graded by four parents, judging their effectiveness in the experimental experience, with the scores set to a five-point system ranging from the most positive (+2) to the most negative (−2).

The experiment process took the form of a “bubble-blowing game” for auxiliary therapeutic for children with autism. Three autistic children are joined by other children hand in hand to form an inner circle, and parents of autistic children form a peripheral circle hand in hand with other children’s parents and interact with the child’s behavior and language. The instructor speaks while doing actions: “blow bubbles, bubbles become bigger, bubbles become smaller, bubbles are broken, and so on.” When the bubble sits big, the children circle outward. When the bubble becomes the hour, the children gather inward in the middle. When the bubble breaks, the children jump up.

**Figure 6:**
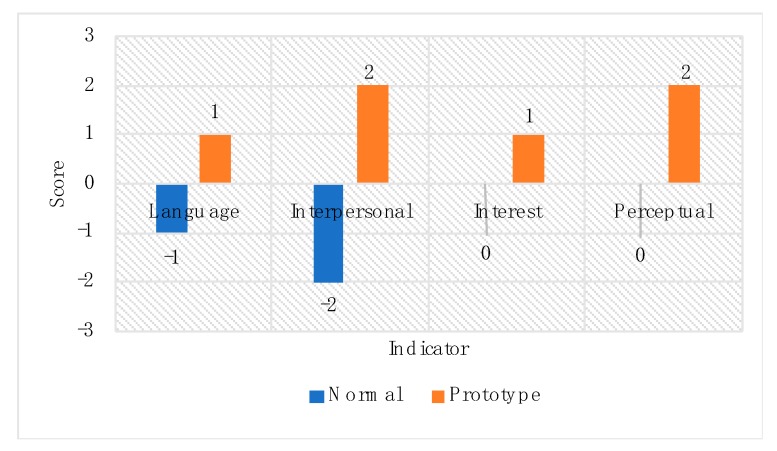
Evaluation of autistic children’ parents.

The four patient’s parents rated the effect of wearing their normal daily clothing and prototype in the experiment, giving a surprisingly consistent score. The four scores of the prototype are significantly higher than that of ordinary daily clothing (Figure 6). This suggests that the actual effect of the prototype in the experimental process has been recognized by the patient’s parents, and the prototype could help improve social communication activities for children with autism, especially for the combination of natural auxiliary treatment such as behavioral therapy, game therapy, and sensory integration therapy. This is a response to Challenges 1 and Q2 mentioned above.

## 6. Humanistic Evaluation and Discussion

The prototype design is not only for autistic children but also for most children’s families. Therefore, more extensive evaluation and analysis should be done. According to the CPCS model, in the process of interactive clothing design, the formation of wisdom needs to reach the cognition level through knowledge mining, that is, humane evaluation. The evaluation approach in this study adopted the Kansei Engineering method [20] rather than ergonomic or functional evaluation methods because this study used parent–child clothing that emphasizes emotion as a research carrier.

### 6.1. Selection of Comparative Evaluation Objects

**Figure 7:**
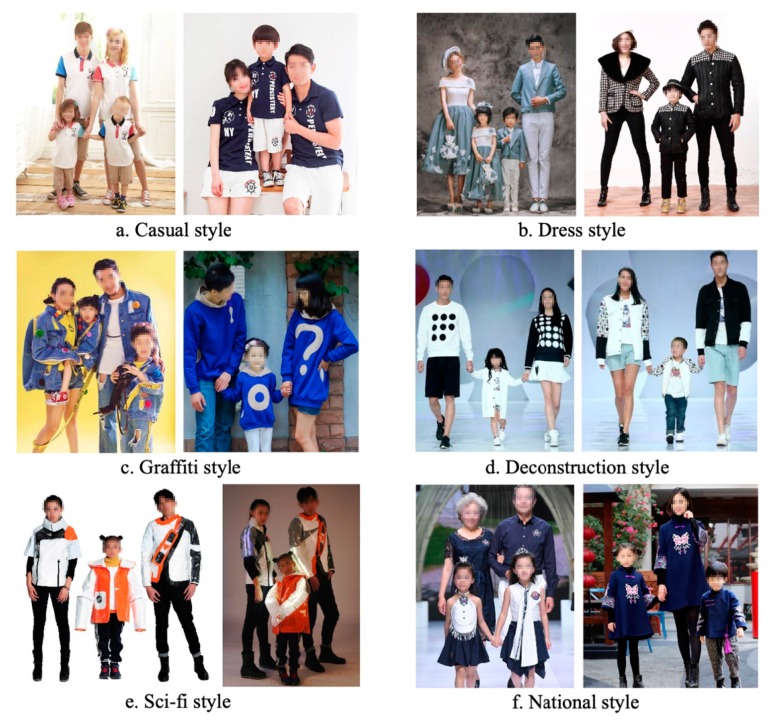
Categories of comparison. (**a**) Casual style, emphasizing a natural and straightforward style; (**b**) Dress style, shows a classical beauty; (**c**) Graffiti style, an extraordinary and exaggerated style; (**d**) Deconstruction style, a concept of structural decomposition; (**e**) Sci-fi style, refers to smart clothing; (**f**) National style, combines the elements of traditional national costumes.

Evaluation elements included Casual style, Dress style, Graffiti style, Deconstruction style, National style, and our prototype (i.e., Optical sensing sci-fi style) (Figure 7 [9]). These six categories could cover and represent the existing types of parent–child clothing on the market.

### 6.2. Selection of Semantic Opposite Adjective

Three categories of words, for a total of 24 pairs of antonym phrases (left of Table 2 [9]), were refined and constructed. These words derive from more than 40 pairs of descriptive words used to evaluate parent–child clothing in our previous interview with professional fashion designers and young parents. A selection was made from the three-level semantic differential concept of parent–child clothing, which was summarized by the prior interview: the clothing style design (I), engineering technology (II), parent–child clothing extension meaning (III).

### 6.3. Participants and Evaluation Methods

There are three groups of participants from China and Japan: clothing industry personnel (20 people), information science or optoelectronic engineer (23 people), and children’s parents (36 people, including four parents of autistic children). The reasons for choosing these groups were as follows: clothing industry personnel, who are specifically engaged in clothing design, could compare clothing to make a professional evaluation. Additionally, because the prototype of this experiment used information sensing technology and optoelectronic technology, it was hoped that the researchers of information engineering and optoelectronic engineering could give a corresponding evaluation from the perspective of the application of intelligent technology. The target purchase group of parent–child clothing is in the parents’ cluster, and thus the result will be more convincing with the help of an evaluation from target users [46].

After providing brief introductions to participants about the different contexts of the six categories of evaluation elements, participants were asked to rate, according to their knowledge background and living habits, each of their perceptions regarding the six categories of parent–child clothes using semantic differential scales. For the evaluation scales, consider the paired item “Smart and Rigid” as an example—it was divided into seven points from the “very rigid” level to the “very smart” level and has assigned scores of −3, −2, −1, 0, 1, 2, 3 respectively. Finally, 73 valid questionnaires were collected, and the other six questionnaires filled out by clothing industry personnel were eliminated due to similarity or lack of credibility.

### 6.4. Differences of Evaluation Results Among the Four Groups

We used the intuitive moving average value method to present the evaluation score differences of four evaluation participants for six samples in Figure 7, especially to focus on the evaluation difference between parents of autistic children and the other three groups.

**Figure 8:**
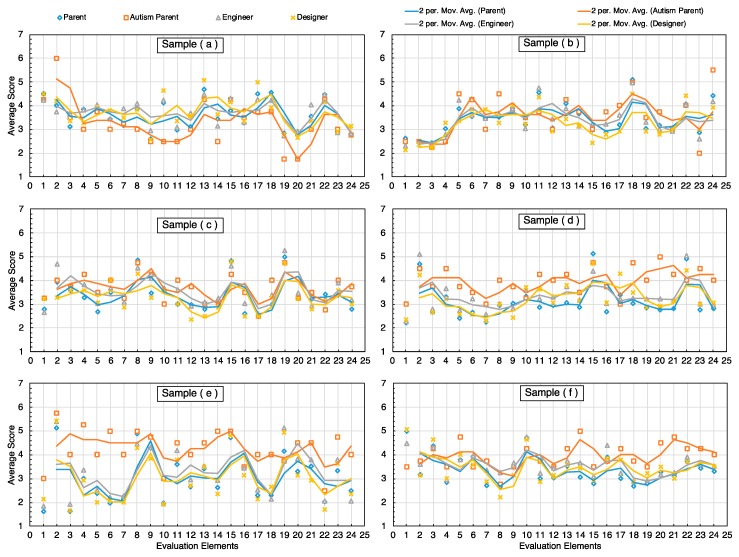
Differences in evaluation results among the four groups. The scoring indexes of each category of clothing from Figure 7 are set to 24 items. The order of 1–24 is given by the order of I-III in Table 2. The data from (**a**–**f**) in Figure 8 correspond to the Sample clothing (**a**–**f**) in Figure 7.

According to Figure 8, we found that parents of autistic children rated Sample (a) significantly lower than the other three groups. The evaluation of Samples (b) and (c) was generally the same for the four groups. The parents of autistic children were slightly higher than the other three groups in the evaluation of Samples (d) and (f). The most significant difference in evaluation between groups was Sample (e), our prototype, in which engineers, designers, and normal children’s parents rated very similar, while parents of autistic children were significantly higher than the other three groups.

This shows that the parents of autistic children fully recognize our thoughts on the sensing interaction design of the prototype and the special function sensing of the prototype for autistic children. Based on the above findings, combined with the results of previous experiments (Figure 6) in the game experience involving parents of autistic children, it can be inferred that the prototype was developed in the right way. However, from Figure 8 Sample (e), we also found that in addition to the parents of autistic children, the overall evaluation of the prototype by three other groups was not high, which indicates that some of the details of the prototype design still needed to be improved and further analysis of the design elements needed to be refined.

### 6.5. Differences between the Prototype and Traditional Parent–Child Clothing

According to the scale statistics on the average values of the evaluation, a comparison between the prototype and the other five categories of the traditional parent–child clothing was made to excavate the difference between the prototype and the traditional parent–child clothing.

According to the 73 participants’ choice of “desire to buy” in all six categories of parent–child clothing, the highest-ranking was the prototype, that is, the Optical sensing sci-fi style (which accounted for 32.9%), followed by the Dress style (which was also the highest-ranking among the five categories of traditional parent–child clothing at 26%). Therefore, the Dress style was chosen as an object for comparison with the prototype.

According to the results of a paired-samples *t*-test between the prototype and traditional parent–child clothing (right of Table 2), 16 of the 24 constructs had significant fixed effects with a *p*-value lower than 0.05. The creativity and other indicators of the prototype were significantly higher than that of the traditional parent–child clothing. However, the disadvantages of the prototype were only the emotional extension elements. There is the most significant difference between the bold and underlined parts of the t value. The constructs corresponding to these values, especially the Concise, Elegant, Systematic, Exquisite, and Warm options, were the weakest points of the prototype but also the critical areas for future development that need to be improved. The Smart, Technology, Cross-border, Multifunctional, and Interactive can represent the advantages and characteristics of the prototype.

**Figure 9:**
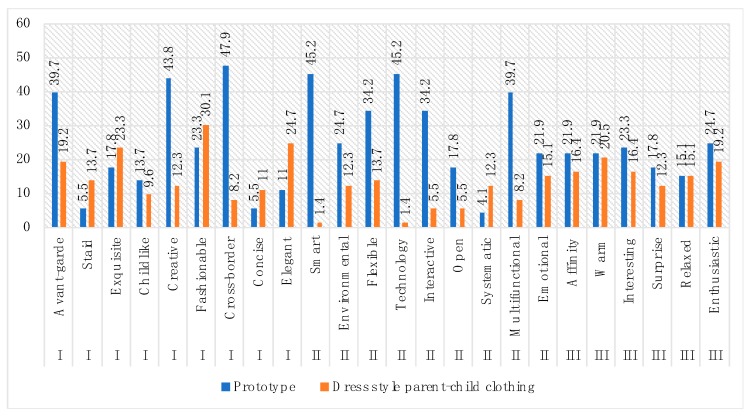
Frequency values of prototype and the Dress style parent–child clothing.

The frequency statistics method was continued to ensure the rationality of the analysis. The five positive semantic adjective items with the highest frequency values of the two categories mentioned above of clothing evaluation were selected as the index of differential analysis (Figure 9). These five indicators were also the characteristics and advantages of the two categories of parent–child clothing. According to the value of the frequency statistic scale, the highest scores of the prototype were Cross-border (47.9%), Smart (45.2%), Technology (45.2%), Creative (43.8%), and Multifunctional (39.7%) while the advantages of the Dress style clothing were Fashionable (30.1%), Elegant (24.7%), Exquisite (23.3%), Warm (20.5%), and Enthusiastic (19.2%), which are also the critical improvement factors of future interactive parent–child clothing development.

## 7. Limitations and Future Work

The prototype in this study is limited to the category of interactive manifestation and does not involve the scope of the big data measurement or the algorithm analysis of the CPS. For the challenges mentioned in the introduction section, that is, the differential needs of different objects, machine learning research needs to be deepened and the algorithm of merging time, task, and scheduling needs to be optimized. The mining and categorization of the basic physical signals and physiological signals of the wearer with autism disorders, as well as the recognition and classification of the social symbols that can be displayed by the clothing, will help to improve the functional development and expand the application areas of the interactive clothing.

## 8. Conclusions

By summarizing the definition of interactive clothing, the CPCS architecture is innovatively constructed and the emergence of successive waves of CPS is proposed that can enable new symbolic interactive ways of coupling the clothes to different wearers. Embedding the CPS can easily achieve interpersonal interaction effects because it embodies people’s feelings. Q1 and Q2 are answered to attain the research objectives to rise to the challenges by bridging the gap between CPS in the field of information science with art and design in the field of humanistic science.

This study summarizes the relationship between CPS architecture and interactive clothing from a humanistic design rather than an information discipline perspective. The CPCS technical implementation model of interactive clothing design under the background of the CPS approach is proposed. Also, the development process of interactive parent–child clothing as a case study for analyzing the transformation of the input of the physical signal to the output of the social symbol recognition in the prototype development process and verifies the feasibility of the CPCS model for interactive clothing design.

The experimental results show that interactive parent–child clothing is not only suitable for the rehabilitation of autistic children but also recognized by most parents. The reasonable embedding of sensing technology can greatly enhance the added value of clothing products. This study provides a fruitful practical application reference for non-information engineers, especially for clothing designers who are engaged in the field of art and design but who are not familiar with the relevant information science and technology. The application principle and technical process of CPCS in the process of interactive clothing development are explained. The satisfactory results of the design and evaluation of prototypes demonstrate that the potential of HCI combined with CPS for developing interactive clothing has just begun, and there are more technical methods and opportunities than ever before to create compelling everyday dress experiences and deep personalization for fashion pioneers as well as researchers devoted in autism rehabilitation.

## Figures and Tables

**Table 1:** Evaluation indicator and content.

No.	Indicator	Content
1	Language expression	Is the child willing to talk to other children or parents compared to participating in daily similar game activities? Is there a logical relationship between every sentence a child says?
2	Interpersonal communication	Does the child tend to have more physical communication with their parents or other children than do similar daily games?
3	Interest expression	Is the child willing to participate in the game?
4	Perceptual communication	Does the child slow to respond to auditory and visual stimuli?

**Table 2:** Semantic opposite adjective pairs and results of the paired-samples T-test.

Category	Semantic		Mean	SD	SE	t	*p*
	Negative	Positive					
I	Conservative	Avant-garde	0.630	1.568	0.184	3.434	0.001
I	Staid	Lively	−0.397	2.559	0.300	−1.326	0.189
I	Rough	Exquisite	−0.753	1.949	0.228	−3.302	0.001
I	Mature	Childlike	0.630	2.010	0.235	2.678	0.009
I	Monotonous	Creative	0.890	1.663	0.195	4.575	0.000
I	Outdated	Fashionable	0.027	1.624	0.190	0.144	0.886
I	Closed	Cross-border	1.411	1.373	0.161	8.782	0.000
I	Tedious	Concise	−1.466	2.109	0.247	−5.939	0.000
I	Vulgar	Elegant	−1.027	1.972	0.231	−4.452	0.000
II	Rigid	Smart	1.781	1.557	0.182	9.774	0.000
II	Destructive	Environmental	0.178	1.743	0.204	0.873	0.386
II	Bound	Flexible	1.068	1.719	0.201	5.312	0.000
II	Traditional	Technology	1.781	1.669	0.195	9.118	0.000
II	Isolated	Interactive	1.192	1.560	0.183	6.526	0.000
II	Closed	Open	−0.137	2.057	0.241	−0.569	0.571
II	Messy	Systematic	−1.082	2.228	0.261	−4.149	0.000
II	Single	Multifunctional	1.808	1.912	0.224	8.079	0.000
II	Rational	Emotional	−0.616	2.271	0.266	−2.319	0.023
III	Alienated	Affinity	0.219	1.652	0.193	1.134	0.261
III	Cold	Warm	−0.603	1.614	0.189	−3.191	0.002
III	Stodgy	Interesting	−0.096	1.108	0.130	−0.740	0.462
III	Disappointed	Surprise	0.164	1.871	0.219	0.751	0.455
III	Gloomy	Relaxed	−0.192	1.883	0.220	−0.870	0.387
III	Indifferent	Enthusiastic	0.521	1.519	0.178	2.927	0.005
